# Structural basis of nectin-1 recognition by pseudorabies virus glycoprotein D

**DOI:** 10.1371/journal.ppat.1006314

**Published:** 2017-05-19

**Authors:** An Li, Guangwen Lu, Jianxun Qi, Lili Wu, Kegong Tian, Tingrong Luo, Yi Shi, Jinghua Yan, George F. Gao

**Affiliations:** 1 Laboratory of Animal Infectious Diseases, College of Animal Sciences and Veterinary Medicine, Guangxi University, Nanning, Guangxi, China; 2 CAS Key Laboratory of Pathogenic Microbiology and Immunology, Institute of Microbiology, Chinese Academy of Sciences, Beijing, China; 3 West China Hospital Emergency Department (WCHED), State Key Laboratory of Biotherapy, West China Hospital, Sichuan University, and Collaborative Innovation Center of Biotherapy, Chengdu, Sichuan, China; 4 CAS Key Laboratory of Microbial Physiological and Metabolic Engineering, Institute of Microbiology, Chinese Academy of Sciences, Beijing, China; 5 College of Animal Science and Veterinary Medicine, Henan Agricultural University, Zhengzhou, Henan, China; 6 National Research Center for veterinary Medicine, High-Tech District, Luoyang, Henan, China; 7 State Key Laboratory for Conservation and Utilization of Subtropical Agro-bioresources, Guangxi University, Nanning, Guangxi, China; 8 Savaid Medical School, University of Chinese Academy of Sciences, Beijing, China; 9 College of Life Sciences, University of Chinese Academy of Sciences, Beijing, China; 10 National Institute for Viral Disease Control and Prevention, Chinese Center for Disease Control and Prevention (China CDC), Beijing, China; 11 Research Network of Immunity and Health (RNIH), Beijing Institutes of Life Science, Chinese Academy of Sciences, Beijing, China; University of California at Los Angeles, UNITED STATES

## Abstract

An early and yet indispensable step in the alphaherpesvirus infection is the engagement of host receptors by the viral envelope glycoprotein D (gD). Of the thus-far identified gD receptors, nectin-1 is likely the most effective in terms of its wide usage by multiple alphaherpesviruses for cell entry. The molecular basis of nectin-1 recognition by the gD protein is therefore an interesting scientific question in the alphaherpesvirus field. Previous studies focused on the herpes simplex virus (HSV) of the *Simplexvirus* genus, for which both the free gD structure and the gD/nectin-1 complex structure were reported at high resolutions. The structural and functional features of other alphaherpesviral gDs, however, remain poorly characterized. In the current study, we systematically studied the characteristics of nectin-1 binding by the gD of a *Varicellovirus* genus member, the pseudorabies virus (PRV). We first showed that PRV infects host cells via both human and swine nectin-1, and that its gD exhibits similar binding affinities for nectin-1 of the two species. Furthermore, we demonstrated that removal of the PRV gD membrane-proximal residues could significantly increase its affinity for the receptor binding. The structures of PRV gD in the free and the nectin-1-bound states were then solved, revealing a similar overall 3D fold as well as a homologous nectin-1 binding mode to its HSV counterpart. However, several unique features were observed at the binding interface of PRV gD, enabling the viral ligand to utilize different gD residues (from those of HSV) for nectin-1 engagement. These observed binding characteristics were further verified by the mutagenesis study using the key-residue mutants of nectin-1. The structural and functional data obtained in this study, therefore, provide the basis of receptor recognition by PRV gD.

## Author summary

Both herpes simplex virus (HSV) and pseudorabies virus (PRV) recognize nectin-1 as the cellular receptor. They utilize the envelope glycoprotein D (gD) on the virion surface to interact with nectin-1, initiating the virus infection. Although the molecular basis of nectin-1 binding by HSV gD has been successfully elucidated with high resolution structures, the atomic features of PRV gD interacting with the same receptor remains uncharacterized. Here, we show that PRV gD exhibits nano-molar affinities for both human and swine nectin-1, and deletion of the membrane-proximal loop in the gD ectodomain dramatically increases its receptor-binding avidity. We further solved the free and the nectin-1-bound PRV gD structures. The free gD structure reveals a canonical fold of an IgV-like core wrapped by the N- and C-terminal extensions as observed in the HSV homologs. The N-terminus of PRV gD, however, is shorter than that of HSV gDs. The solved complex structure demonstrates that PRV gD exhibits a homologous receptor-binding mode to the HSV counterpart. Nevertheless, several unique features at the PRV gD binding interface suffice the viral ligand to engage nectin-1 with a series of residues differing from the HSV amino acids. These observations not only delineated the molecular basis of PRV engaging nectin-1 but also enriched our knowledge on the receptor-binding mechanism of the *Alphaherpesvirinae* subfamily.

## Introduction

There are three major subfamilies, *Alpha-*, *Beta- and Gamma-herpesvirinae*, in the *Herpesviridae* family [1]. The three subfamilies differ in their host range capacities. In contrast to beta and gamma herpesviruses which exhibit restricted or limited cell-type tropism, the alphaherpesviruses have a much broader host range and are able to infect a wide variety of cell types [2, 3]. For example, the representative alphaherpesvirus, herpes simplex virus (HSV), shows low species specificity and is able to infect human and non-human cells alike [4]. The capability of HSV to infect most human cell types is recognized as an important contributing factor to its high prevalence in the world populations [5]. Pseudorabies virus (PRV), another member of the *Alphaherpesvirinae* subfamily, is reported to infect both farming (e. g. pigs, sheep, etc) and pet (e. g. cats) animals [6]. Herpes B virus, an alphaherpesvirus that causes mild or asymptomatic infections in macaques, can cross the species barriers and lead to fatal diseases in humans [7].

Alphaherpesviruses contain multiple surface glycoproteins (e. g. more than 11 in HSV) in the virion envelope [8]. An early step in the infection of the alphaherpesvirus is the engagement of host receptors by the virus glycoprotein D (gD) [5]. The broad tropism of alphaherpesvirus is, therefore, at least partially the result of its capacity to recognize and bind multiple cell surface molecules via gD [8]. Thus far, six gD receptors have been identified. These include 3-O-sulfonated-heparan sulfate (3-O-S-HS) [9]; the herpes virus entry mediator A (HveA, also known as HVEM), a TNF receptor-related protein [10]; and three immunoglobulin superfamily members: HveB (PRR2, nectin-2) [11], HveC (PRR1, nectin-1) and HveD (PRV, CD155) [12]. Of these renowned host molecules, nectin-1 serves as a broadly used receptor mediating the entry of all of the commonly tested viruses, including HSV type 1 (HSV-1) and type 2 (HSV-2), PRV, and bovine herpes virus type 1 (BHV-1) [12–14]. In addition, nectin-1 was also identified as the primary receptor for the HSV-1 infection of rat and mouse sensory neurons [15].

Nectin-1 is an immunoglobulin (Ig)-like cell adhesion molecule [16]. It plays important roles in organizing the intercellular junctions by self homodimerization and/or by heterodimerization with other nectin and nectin-like molecules (e. g. nectin-3, nectin-4, nectin-like 1, etc.) [17]. Nectin-1 is highly conserved in mammalian animals. The swine and human nectin-1 share 96% amino acid identity, and both homologs are able to mediate the entry of HSV-1, HSV-2, PRV and BHV-1 [13, 18]. It is demonstrated that the gD proteins of these alphaherpesviruses bind to nectin-1 with nanomolar affinities [13, 19]. The detailed binding mode between HSV gD and human nectin-1 has been successfully illustrated in several previous studies [20–22]. The gD molecule is composed of a V-set Ig-like (or IgV-like) core and long N- and C-terminal extensions, It utilizes both terminal-extension elements to interact with nectin-1. The receptor, with three Ig-like domains arranged into a rod-shaped structure, projects its membrane-distal IgV domain for gD engagement[21–23]. It is notable that the whole gD footprint in nectin-1 overlaps extensively with the nectin-1 dimerization interface, which explains a novel mechanism of exploiting the host cell-adhesion functions by HSV for virus spread and infection [21, 22].

In addition to tethering virus particles onto cell surface and facilitating the viral spread, gD is also believed to play a key role in triggering the membrane fusion cascade, thereby leading to the entry of alphaherpesviruses [24–26]. Several previous studies demonstrated that gD binding to its receptors could displace the C-terminal pro-fusion domain (PFD) to activate the fusion executor composed of glycoproteins B, H and L (or gB, gH and gL) [24, 25]. Interestingly, the structures of HSV gD in the receptor bound forms (with nectin-1 and HVEM, respectively) and a dimeric free gD structure indeed reveal that receptor engagement would displace the gD C-terminal loop [20–22, 25, 27]. These structural investigations pave the way for understanding the basis of gD recognition of multiple cellular receptors and thereby of the broad cell tropism of alphaherpesviruses. Nevertheless, the previous studies are almost exclusively based on HSV gDs. The structural and functional features of other alphaherpesviral gDs remain poorly characterized. These are interesting scientific issues awaiting answers in the herpesvirus field, because gD homologs of the prevalent alphaherpesviruses (e. g. HSV, PRV, BHV-1, etc.) only share very low (22–33%) amino acid sequence identities [19].

To further delineate the receptor recognition basis of alphaherpesviruses, we set out to investigate the detailed interactions between PRV gD and nectin-1. Despite the significant homology between PRV and HSV, the two viruses belong to different genera (PRV in the *Varicellovirus* genus and HSV in the *Simplexvirus* genus, respectively [1]). In the current study, we show that PRV infects host cells via both human and swine nectin-1, but not with human HVEM. PRV gD engages the human and swine receptors with similar affinities. We further demonstrate that a gD variant lacking the C-terminal membrane-proximal residues exhibited much higher affinity for nectin-1, which we believe represents an inspiring evidence that the PRV gD C-terminal loop likely also plays a role in triggering the virus/cell membrane fusion as observed for HSV PFD. The structures of PRV gD in the unbound and the nectin-1-bound forms were then solved at high resolutions. The PRV protein exhibits overall similar 3D fold and nectin-1 binding mode to its HSV counterpart but utilizes different gD residues (from those of HSV) for nectin-1 engagement. Finally, we also conducted a mutagenesis study using the interface-residue mutants of nectin-1, which in combination with the structural observations, provide the basis of receptor recognition by PRV gD.

## Results

### PRV utilizes both human and swine nectin-1, but not HVEM, for cell entry

**Fig 1:**
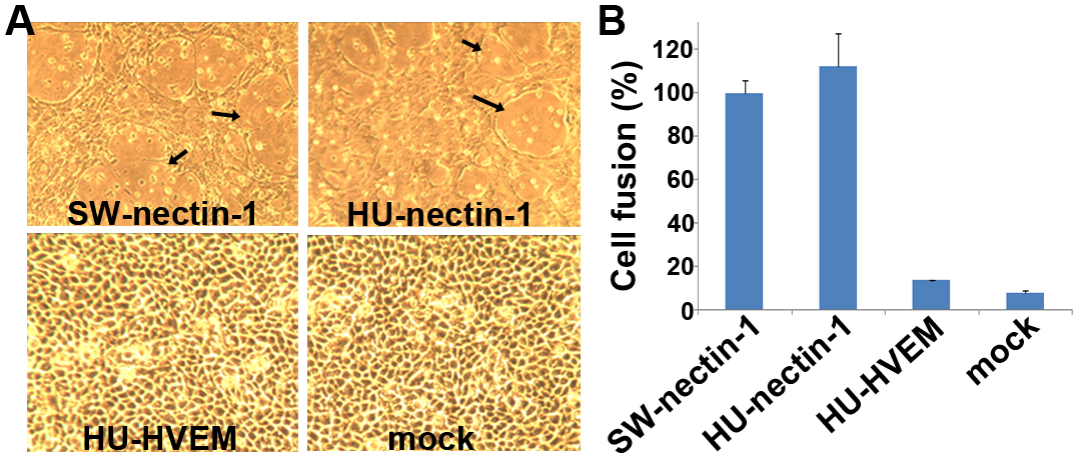
PRV recognizes both human (HU-nectin-1) and swine (SW-nectin-1) nectin-1 for cell entry. (A) PRV infects cells expressing HU- and SW-nectin-1, but not cells expressing human HVEM (HU-HVEM). CHO-K1 cells were transfected for transient expression of HU-, SW-nectin-1 and HU-HVEM, and subsequently infected with a PRV vaccine strain. Clear cytopathic changes were observed with HU- and SW-nectin-1, which are marked with arrows. (B) HU- and SW-nectin-1 mediate the gD-dependent cell fusion. A cell-based fusion assay was set up with CHO-K1 cells as previously applied in the HSV studies[21, 22]. CHO-K1 cells expressing PRV gD/gB/gH/gL and T7 luciferase were mixed and incubated with those expressing T7 polymerase in combination with HU-/SW-nectin-1 or HU-HVEM. The resultant luciferase activity was calculated and quantitatively compared with that observed for SW-nectin-1 (determined as 100% cell fusion). The results of three independent experiments were histogrammed as the means ± standard deviations (SD).

Of the thus-far identified gD receptors, nectin-1 has been shown to mediate the cell entry of multiple alphaherpesviruses [5, 8, 12]. Several previous studies demonstrated that both human (HU-nectin-1) and swine nectin-1 (SW-nectin-1) serve as a cellular receptor for PRV [13, 28]. As a step towards understanding the basis of receptor recognition by the virus, we first reconfirmed the nectin-1 mediated viral entry using the PRV vaccine strain Barth K61 (28) in CHO-K1 cells. CHO-K1 cell lacks any of the known alphaherpesvirus receptors and is therefore resistant to PRV infection [29]. Transient expression of HU- and SW-nectin-1 in CHO-K1, however, suffices the cells for PRV entry. In the presence of either the human or the swine receptors, PRV infection with significant cytopathic effects was observed; whereas HU-HVEM failed to promote the entry of the virus (Fig 1A). This is consistent with previous reports showing that nectin-1, but not HVEM or 3-O-S-HS, could mediate the PRV infection [8].

To quantitatively compare the cell entry mediated by HU- and SW-nectin-1, we further set up a cell based fusion assay as previously applied in the HSV studies [22]. With CHO-K1 cells, remarkable cell fusion could be observed when the nectin-1-expressing cells are mixed with cells expressing HSV gD along with the viral fusion executor of gB, gH and gL. By replacing the HSV glycoproteins with the PRV homologs, significant increase in the luciferase activity was recorded, which is highly indicative of fusion of the cells. On the whole, the cell fusion mediated by HU- and SW-nectin-1 is quantitatively equivalent (Fig 1B), indicating similar capacities of gD engagement by the two receptors.

### PRV gD engages human and swine nectin-1 with a similar binding affinity

**Fig 2:**
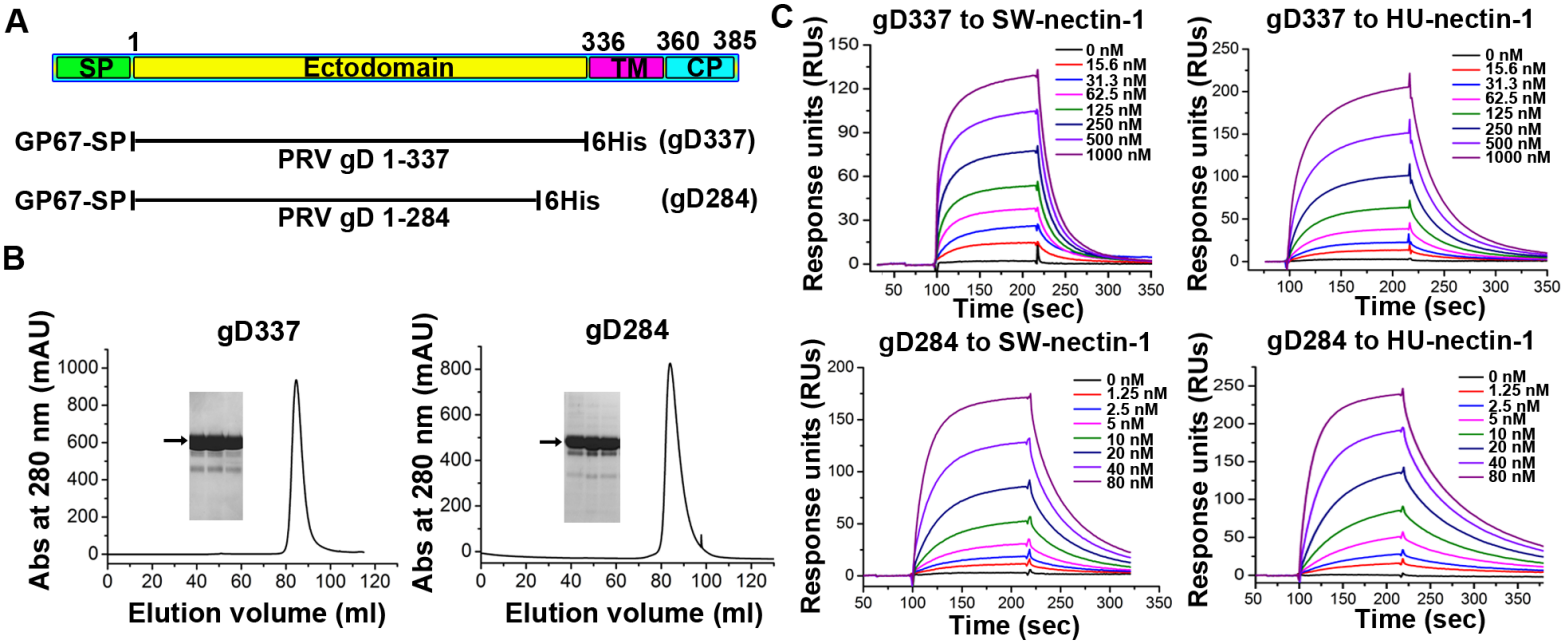
An intimate binding between PRV gD and HU-/SW-nectin-1. (A) A schematic picture of PRV gD. The boundaries of the domain elements, including the signal peptide (SP), the ectodomain, the transmembrane domain (TM), and the cytoplasmic domain (CP), were determined by bioinformatic predictions using the SignalP4.1 and TMHMM web-server. For recombinant expression of PRV gD in insect cells, the protein was truncated (aa 1–337 for gD337 and aa 1–284 for gD284), engineered with a GP67 signal peptide for secretion, and added with a C-terminal 6His tag for purification. (B) Representative size-exclusion chromatographs of gD337 and gD284. The recombinant PRV gD proteins were purified from the supernatants of the baculovirus-infected insect cells, analyzed on a Hiload 16/60 Superdex 200 column (GE), and then examined by electrophoresis by SDS-PAGE. The resultant separation profiles are shown. (C) An SPR assay characterizing the PRV-gD/nectin-1 binding kinetics. Gradient concentrations of gD337 or gD284 were flow through SW- and HU-nectin-1 immobilized on the chip surface. The real-time binding profiles are recorded and shown.

To gain further insight into the PRV-gD/nectin-1 interaction, we set out to characterize their binding features using the real-time surface plasmon resonance (SRP) assays. Previous studies have shown that, by truncation of the C-terminal trans-membrane and cytoplasmic domains, the entire ectodomain of PRV gD could be yielded as a recombinant protein in the soluble form [13]. Following the same strategy, we successfully expressed and purified a truncated PRV gD protein spanning residues 1–337 (hereafter referred to as gD337) using a baculovirus expression system (Fig 2A and 2B). Noted that the residue numberings for HSV gD in previous structural studies are based on the mature protein [21, 22], the numberings for PRV gD amino acids in the current study are therefore also based on the mature protein (unless otherwise specified) to facilitate sequence comparison (Fig 2A). The ecto-domain proteins of both HU- and SW-nectin-1 prepared from *E*. *coli* were individually immobilized on the chip and tested for the interaction with gD337. As expected, the gD protein potently interacts with both receptors, exhibiting typical slow-on/slow-off binding kinetics (Fig 2C). The equilibrium dissociation constants (*K*_d_) were determined to be 191 nM with HU-nectin-1 (*K*_on_, 1.41 × 10^5^ M^-1^s^-1^; *K*_off_, 2.7 × 10^−2^ s^-1^) and 301 nM with SW-nectin-1 (*K*_on_, 1.46 × 10^5^ M^-1^s^-1^; *K*_off_, 4.4 × 10^−2^ s^-1^), respectively. Therefore, PRV gD recognizes the human and swine receptors with essentially the same binding affinity. This result is consistent with our observation in the cell fusion assay which shows that nectin-1 of the two species mediates gD-dependent CHO-K1 fusion with similar efficiencies shown in the previous section. Furthermore, the determined *K*_d_ values are also in good accordance with a previous study reporting an affinity of approximate 130 nM for the PRV-gD/HU-nectin-1 interactions [13].

### Removal of the PRV gD membrane-proximal residues increases its affinity for nectin-1

Inspired by the studies on HSV gD which revealed an approximate 2-digit fold affinity difference in receptor binding between a long and a short (lacking about 20 residues at the membrane-proximal region) gD form [20, 25], we further constructed a shorter PRV gD variant without the membrane-proximal loop. This new construct spans the gD amino acids 1–284 (hereafter referred to as gD284) (Fig 2A). The resultant protein was similarly prepared via the baculovirus expression system and purified to homogeneity (Fig 2B). When tested using SPR, this short form of PRV gD exhibited significantly increased binding avidity to nectin-1. The calculated *K*_d_s of gD284 to the human and swine receptors were 16.1 nM (*K*_on_, 7.25 × 10^5^ M^-1^s^-1^; *K*_off_, 1.17 × 10^−2^ s^-1^) and 18.4 nM (*K*_on_, 1.15 × 10^6^ M^-1^s^-1^; *K*_off_, 2.11 × 10^−2^ s^-1^), respectively (Fig 2C). These values represent an approximate 12–16 fold enhancement in affinity in comparison to gD337. It is notable that gD284, with the enhanced receptor binding capacity, exhibits a similar affinity for nectin-1 to HSV-1 gD285—a representative short form of HSV gD [21, 22].

According to the previous studies, the observed affinity-difference between the long and short forms of HSV gD stem mainly from the changes in their kinetic association rates (up to 40-fold in *K*_on_) [25]. The enhanced affinity for nectin-1 by PRV gD284 (relative to gD337), however, arises from both the increase of the association rate (by approximate 5–8 fold in *K*_on_) and the decrease of its dissociation rate (by approximate 2-fold in *K*_off_).

### The structure of free PRV gD

To probe into the structural features of PRV gD, both gD337 and gD284 were subjected to intensified crystallization screenings. We finally managed to collect a 1.5-Å resolution data set from the gD337 crystals. The solved structure was refined to *R*_work_ = 0.169 and *R*_free_ = 0.192 (Table 1), and contains 244 amino acids spanning from P7 to P250. The terminal residues (A1 to V6 in the N-terminus and R251 to S337 in the C-terminus), however, were untraceable. We believe these two parts, especially the large C-terminal region, are flexible and most likely disordered loops, which might have undergone unexpected proteolytic digestions during crystallization.

**Table 1:** Data collection and refinement statistics.

	gD337	gD284/SW-nectin-1
**Data collection**		
Space group	C2	P212121
Cell dimensions		
*a*, *b*, *c* (Å)	79.40, 60.98, 60.68	79.95, 98.11, 128.40
α,β,γ (°)	90.00, 111.22, 90.00	90.00, 90.00, 90.00
Resolution (Å)	50–1.50 (1.55–1.50)	50–2.70 (2.80–2.70)
*R*_sym_ or *R*_merge_	0.062 (0.299)	0.228 (1.187)
*I* / σ*I*	25.5 (7.3)	9.6 (1.8)
Completeness (%)	97.6 (97.0)	99.9 (100.0)
Redundancy	6.1 (6.3)	8.6 (8.7)
**Refinement**		
Resolution (Å)	23.53–1.50	38.17–2.70
No. reflections	42206	28251
*R*_work_ / *R*_free_	0.1691/0.1923	0.2379/0.2664
No. atoms		
Protein	1933	5457
Ligand/ion	0	0
Water	334	85
*B*-factors		
Protein	23.0	46.9
Ligand/ion		
Water	36.5	40.1
R.m.s. deviations		
Bond lengths (Å)	0.006	0.015
Bond angles (°)	1.090	1.512

Values in parentheses are for highest-resolution shell.

**Fig 3:**
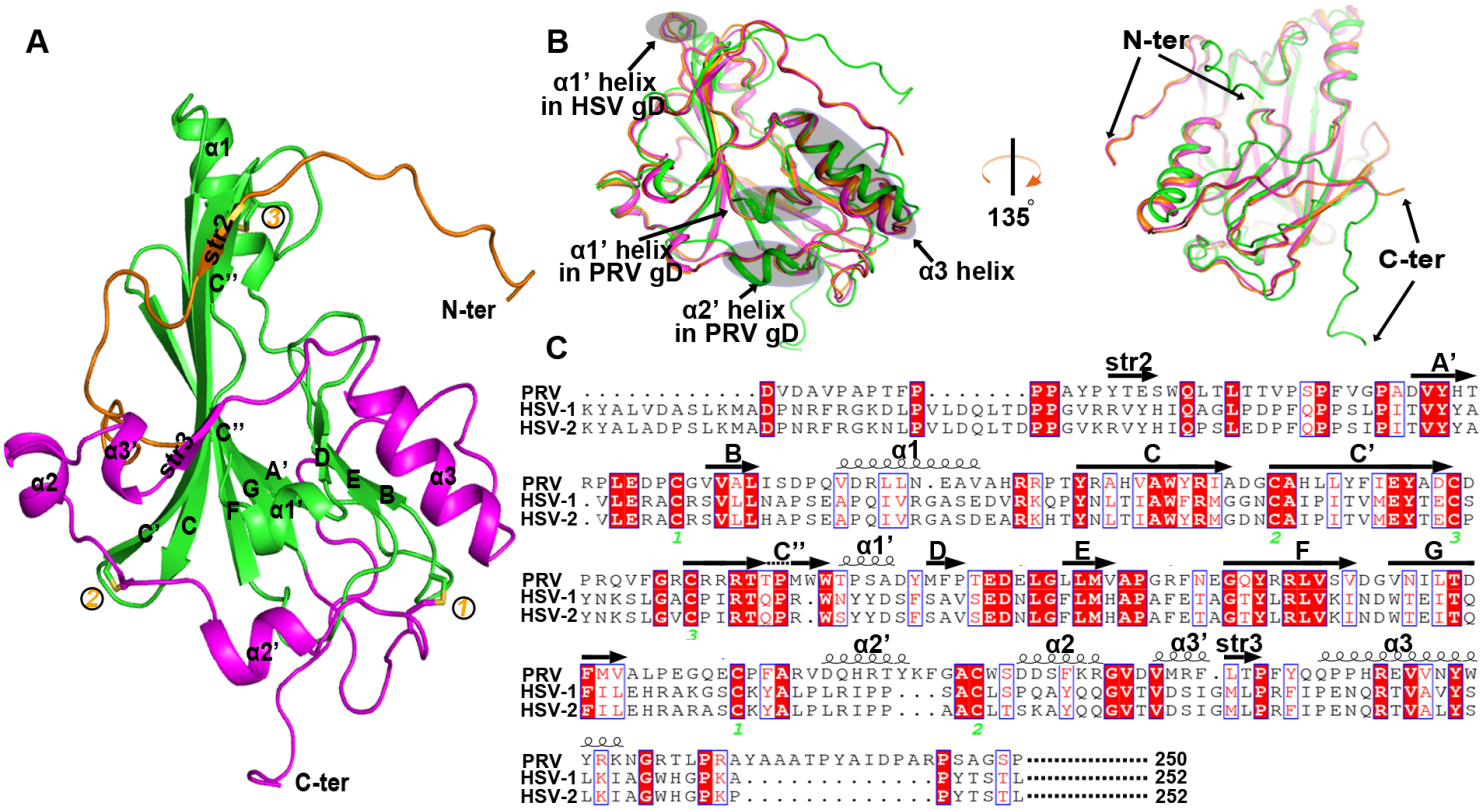
Structure of the unbound PRV gD. (A) Cartoon representation of the overall structure. The PRV gD structure is composed of an IgV-like core and the surface-exposed N- and C-terminal extensions, which are colored green, orange, and magenta, respectively. The secondary elements referred to in the text are labeled. The three disulfide bonds and the N- and C-termini are marked. (B) Superimposition of the PRV (green), HSV-1 (orange), and HSV-2 (magenta) gD structures. The shaded circles and the arrows mark the variant secondary structure elements and the significant conformational differences between PRV gD and its HSV homologs. The right panel is rotated along a vertical axis for about 135 degrees to highlight the large orientation variance observed for the terminal loops. (C) A structure-based sequence alignment for PRV gD and its HSV homologs. The spiral lines and the horizontal arrows indicate α-helices and β-strands, respectively. The conserved cysteine residues that form disulphide bonds in both the PRV and the HSV gD structures are highlighted and marked numerically. For clarity, sequences of the signal peptide sequence, the C-terminal membrane-proximal loop, and the transmembrane and cytoplasmic domains were not included for the comparison. The residue numberings are based on the gD sequences of the mature proteins.

The overall PRV gD structure is expectedly composed of an IgV-like core and the long N- and C-terminal extensions (Fig 3A), as observed in its HSV homologs [20–22]. The core-domain, which spans from D38 to V164, contains a nine-stranded (A', B, C, C', C'', D, E, F, and G) β-barrel in the center. These nine strands are topologically arranged in a typical IgV manner. In contrast to the canonical IgV fold, however, the barrel C'' strand is kinked in the middle, leading to two strand halves connected with a distorted loop. Similar kinked C'' strand has also been observed in both HSV-1 and HSV-2 gD structures, which likely represents a unique feature of the alphaherpesviral gDs. In addition to the compact central barrel, the core-domain also contains two α-helices (α1 and α1'), locating in between the BC and the C''D strands, respectively (Fig 3A).

The IgV-like core of PRV gD is further wrapped by the N- and C-terminal extensions. The former, extending from P7 to A37, is largely a loop structure. It also encompasses a small β-strand (str2) which is aligned in parallel with the first half of strand C''. The latter extension, with residues A165-P250, is much more extended. It structurally folds into four α-helices (α2', α2, α3' and α3) and one small β-strand (str3), covering about half of the central-core outer surface (Fig 3A). In total, three disulfide bonds were formed in the PRV gD structure. One (C100 with C109) is observed in the IgV-like core, connecting the C' and C'' strands; while the other two (C49 with C172 and C88 with C188, respectively) are located between the central core and the C-terminal extension, tying the extension loops to the core components. These disulfide bridges are also conserved in HSV gDs (Fig 3C).

As expected, the structure of PRV gD is quite similar to those of its HSV homologs. Superimposition of our structure with previously reported HSV gD structures revealed well-aligned core domain and terminal extensions (Fig 3B). Despite of the low sequence identity, a majority of the secondary structure elements, including the core-strands and most of the extension helices, were parallel-formed in PRV and HSV gDs. The PRV structure, however, contains an extra α2' helix in the C-terminal extension. In addition, while both PRV and HSV gDs encompass a core α1' helix, their steric positions are quite different in the structures. In PRV gD, helix α1' is located between strands C'' and D; whereas the HSV-gD α1' helix lines before strand C, directly following the α1 helix (Fig 3B and 3C). Further structural variations between PRV and HSV gDs were observed for the equivalent α3 helices and their terminal loops. The former showed a small variance in the conformation, while the latter exhibited large orientation differences (Fig 3B). Facilitated by the structure-based sequence alignment, it is notable that HSV gD encompasses a much longer N-terminal loop (or N-loop) than PRV gD (Fig 3C). Noted that HSV gD reconstitutes its N-loop into a hairpin structure for HVEM binding [27], the lack of an N-loop of sufficient size in PRV gD therefore coincides well with its incompetence of utilizing HVEM.

### The structure of PRV gD bound with swine nectin-1

Inspired by the high affinity between gD284 and nectin-1, we prepared the complex of this short form of PRV gD bound with the IgV-domain protein of the SW-nectin-1, and obtained a complex crystal that diffracts to 2.7 Å resolution. The complex structure, with an *R*_work_ of 0.238 and an *R*_free_ of 0.266 (Table 1), contains gD residues P9-D241 and the nectin-1 amino acids D37-M143. Although a shortened gD form was used for the complex crystallization, a large fraction of the C-terminal residues were still untraceable in the structure. It is notable that similar proportion of the gD residues were successfully traced in both the free gD and the gD/nectin-1 complex structures, despite that two different gD forms (gD337 and gD284, respectively) were individually used in the crystallization experiments.

**Fig 4:**
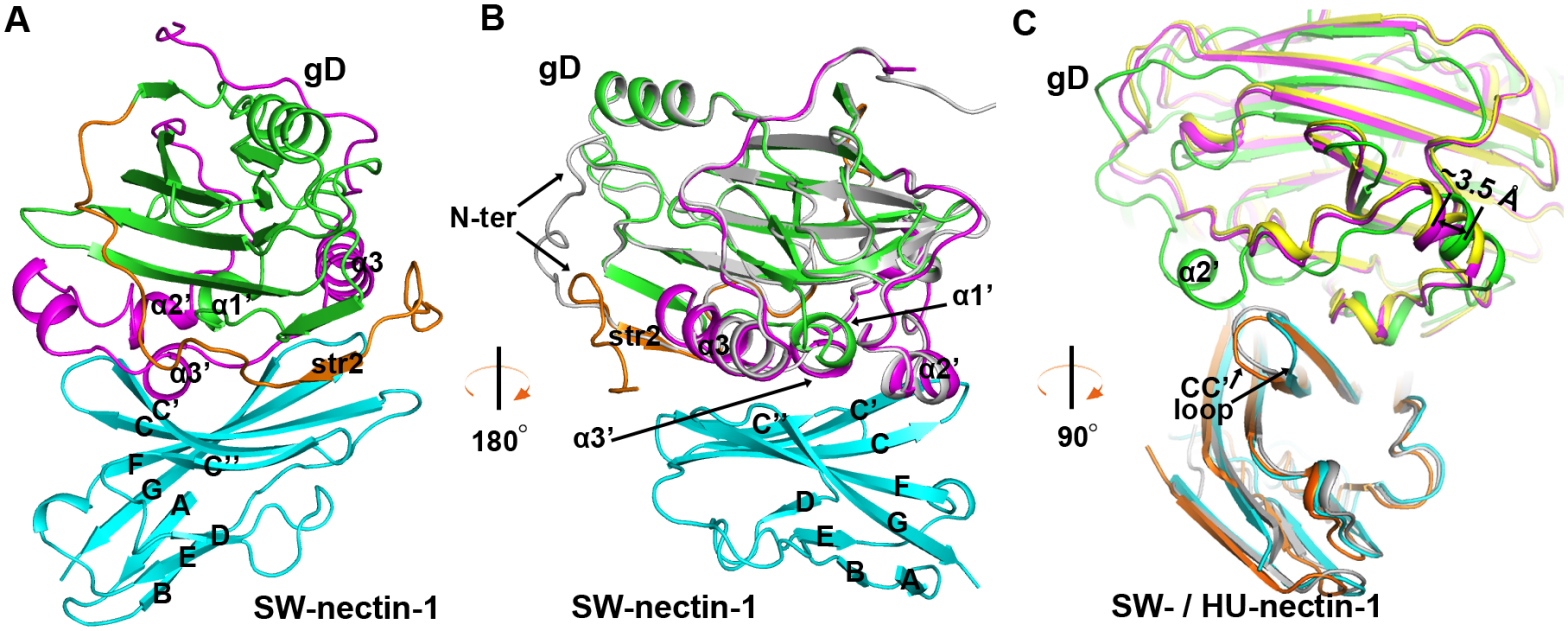
Structure of the PRV-gD/SW-nectin-1 complex. (A, B) Cartoon representation of the overall structure. The gD molecule is colored as in Fig 3A, and the membrane-distal IgV domain of SW-nectin-1 is shown in cyan. Those elements referred to in the text, including the secondary structure elements of SW-nectin-1 IgV and the interface elements in PRV gD, are labeled. The free PRV gD structure (in gray) was also aligned to the complex structure in (B) to highlight the reorientation of the gD N-terminal loop upon receptor binding. (A) The complex structure of PRV gD bound to SW-nectin-1. (B) The same complex structure that is shown after horizontal rotation of about 180 degrees. (C) Comparison of the PRV-gD/SW-nectin-1 (PRV gD in green and SW-nectin-1 is cyan) complex structure with previously reported HSV-1-gD/HU-nectin-1 (HSV-1 gD in yellow and HU-nectin-1 in orange) and HSV-2-gD/HU-nectin-1 (HSV-2 gD in magenta and HU-nectin-1 in gray) complex structures. The CC' loop of variant conformations in nectin-1, the 3.5 Å shift between the bound PRV and HSV gDs, and the unique α2' helix in PRV gD bulged towards the CC' loop of nectin-1 are highlighted and labeled. For clarity, the view of the structure in panel (C) is clockwise rotated along the vertical axis for about 90 degrees relative to that in panel (B).

As expected, the gD molecule in the receptor-bound state similarly folds as an IgV-like core wrapped by the extensive terminal extensions. It recognizes SW-nectin-1 mainly through the extension elements (including str2 and its flanking loops in the N-terminal extension, and the α2', α3', α3 helices, and the α3'/α3 intervening loop in the C-terminal extension) and the exposed α1' helix of the core domain (Fig 4A and 4B). These gD components were exquisitely positioned over the binding interface, engaging exclusively the V-domain CC'C''FG sheet of the receptor (Fig 4A and 4B). In comparison to the free PRV gD structure, its N-loop was clearly reoriented upon receptor binding, extending to the vicinity of helix α3 in the complex structure (Fig 4B). Apart from this N-loop re-orientation, engagement of SW-nectin-1 does not induce other significant conformational changes in the viral ligand.

Overall, the nectin-1 binding mode of PRV gD resembles its HSV counterparts. All the three viral ligands utilize the terminal extensions to contact the CC'C''FG sheet of the receptor IgV domain. A detailed superimposition between the current structure and the previously reported HSV-gD/nectin-1 complex structures, however, revealed an obvious difference in their steric position (relative to the receptor) for the bound gD proteins. With well-aligned nectin-1 molecules, a shift-distance of about 3.5 Å was observed between the PRV and HSV gDs (Fig 4C). Furthermore, the receptor CC' loop also showed quite different conformations when bound to the viral ligands of PRV and HSV (Fig 4C). Noted that PRV gD contains an extra α2' helix which is projected outwards for engagement of the receptor, the SW-nectin-1 CC' loop therefore adopted an alternative conformation to accommodate the bulged helix. Apart from the aforementioned differences, the PRV and HSV gDs exhibited quite similar overall-receptor-binding mode for engagement of nectin-1 (Fig 4C).

### Structural basis of the nectin-1 recognition by PRV gD

**Fig 5:**
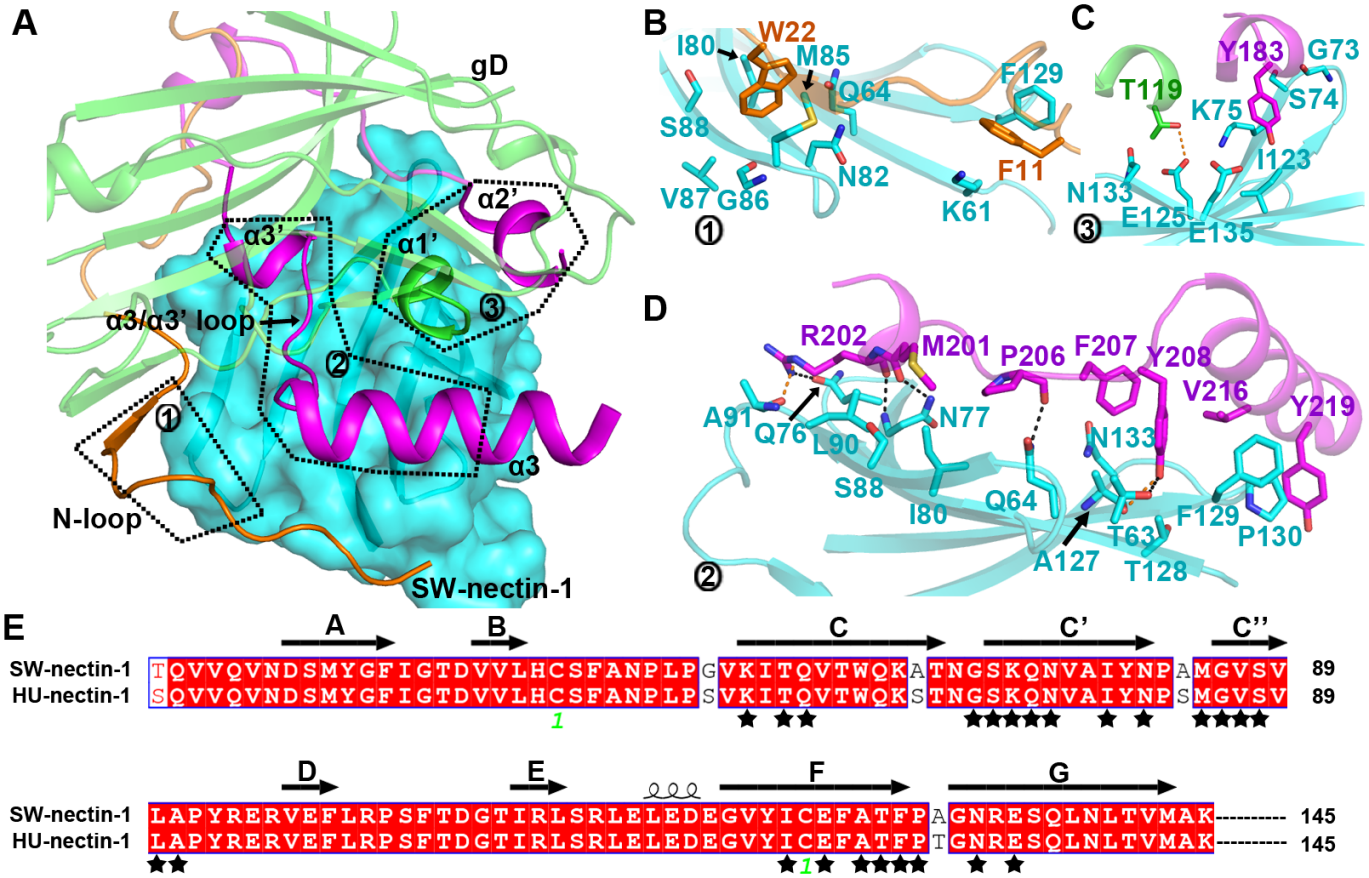
The atomic interaction details at the PRV-gD/SW-nectin-1 interface. (A) An overview of the binding interface. The nectin-1 receptor is shown in surface and the gD ligand is presented as ribbons. The interface components in PRV gD, including the N-loop, the α1' helix in the IgV-like core, and the α2', α3', α3 helices and the α3'/α3 intervening loop in the C-terminal extension, are highlighted and marked with patch numbers 1–3. The amino acid interaction details for each of the three patches were delineated in panels (B), (C), and (D), respectively. (B) The interaction of the gD N-loop with nectin-1. (C) The interaction of the gD α1' and α2' helices with nectin-1. (D) The interaction of the gD α3', α3 helices and their intervening loop with nectin-1. The residues referred to in the text are shown and labeled. Dark dashed lines indicate strong H-bonds (<3.0 Å), while orange ones represent weak H-bonds (3.0–3.5 Å). (E) Amino acid sequence alignment between HU- and SW-nectin-1 highlighting their IgV domains that are recognized by PRV gD. The residues interfacing with gD are marked with black stars. For clarity, only those that contribute >2 inter-molecule Van der Waals contacts were selected. A full list of the interface residues were summarized in Table 2.

On the whole, extended surface areas of about 1118.1 Å^2^ in PRV gD and 1170.9 Å^2^ in SW-nectin-1 were buried upon complex formation. We therefore scrutinized this binding interface to delineate the amino acid interaction details between the two binding entities. Along the nectin-1 CC'C''FG sheet, the major receptor-engagement components of gD include its N-loop, the α1' helix in the IgV-core, and helices α2', α3', α3 and the α3'/α3 loop in its C-terminal extension (Fig 5A). In the N-loop, two aromatic residues F11 and W22 were projected outwards for receptor binding. They were found to be packed against nectin-1 amino acids K61, Q64, I80, N82, M85-S88, and F129, thereby providing important hydrophobic interactions (Fig 5B). It should be noted that F11 of gD is located in the N-loop region that was shown to undergo large conformational changes after receptor binding (Fig 4B). We believe the subsequent interactions of F11 with nectin-1 K61 and F129 are the major forces stabilizing the observed N-loop orientation in the complex structure. As for the α1' and α2' helices, each component presents one amino acid (T119 in α1' and Y183 in α2') towards the receptor, contacting mainly the nectin-1 residues G73-K75, I123, E125, N133, and E135 via multiple Van der Waals (vdw) interactions. In addition, the α1'-residue T119 also forms a weak H-bond with nectin-1 E125 (Fig 5C). Further PRV-gD/SW-nectin-1 interactions were contributed by the α3', α3 helices and their intervening loop, which position multiple residues, including M201-R202, P206-Y208, V216, and Y219, to interact with nectin-1 amino acids T63-Q64, Q76-N77, I80, S88, L90-A91, A127-P130 and N133 (Fig 5D). In addition to providing extensive vdw contacts, these amino acids further stick the viral ligand to the receptor by creating an inter-molecule H-bond network. In total, five strong and two weak H-bonds were observed to form, arising from PRV gD M201 and R202 interacting with SW-nectin-1 Q76, N77 and A91, gD P206 with nectin-1 Q64, and gD Y208 with nectin-1 T63 and A127, respectively (Fig 5D). It is notable that the identified interface residues in the receptor are absolutely conserved between HU- and SW-nectin-1 (Fig 5E), explaining the similar affinities of the two receptors for PRV gD binding.

Owing to the low sequence identity, PRV and HSV gDs utilize different amino acids for nectin-1 engagement. Comparison of their footprints in nectin-1, however, reveals essentially the same surface patch in the receptor. For both PRV and HSV gDs, the nectin-1 residues locating within a distance of 4.5 Å from the bound ligand were selected and compared in detail. The subsequent interface-residue list revealed 31 amino acids interacting with PRV gD, 22 with HSV-1 gD and 26 with HSV-2 gD, respectively. Of these residues, 20 were utilized for contacting all the three viral ligands, contributing more than 90% of the total inter-molecule contacts with either ligand (Table 2). Though the vdw contacts conferred by each of these amino acids were different between HSV and PRV gDs (Table 2), these shared 20 nectin-1 residues dominate the binding to gDs of both viruses.

**Table 2:** Comparison of the nectin-1 residues contacting PRV gD with those interfacing with HSV-1 and -2 gDs^a^.

Nectin-1 in the PRV-gD/SW-nectin-1 complex	Nectin-1 in the HSV1-gD/HU-nectin-1 complex	Nectin-1 in the HSV2-gD/HU-nectin-1 complex
——	S59 (1 contact)	S59 (2 contacts)
**K61 (8 contacts)**	**K61 (10 contacts)**	**K61 (8 contacts)**
**T63 (10 contacts)**	**T63 (15 contacts)**	**T63 (5 contacts)**
**Q64 (20 contacts)**	**Q64 (19 contacts)**	**Q64 (13 contacts)**
**T66 (1 contact)**	**T66 (1 contact)**	**T66 (3 contacts)**
**Q68 (1 contact)**	**Q68 (6 contacts)**	**Q68 (12 contacts)**
A70 (1 contact)	——	——
G73 (5 contacts)	——	G73 (4 contacts)
S74 (11 contacts)	——	S74 (3 contacts)
**K75 (24 contacts)**	**K75 (7 contacts)**	**K75 (13 contacts)**
**Q76 (15 contacts)**	**Q76 (10 contacts)**	**Q76 (14 contacts)**
**N77 (29 contacts)**	**N77 (35 contacts)**	**N77 (42 contacts)**
**I80 (11 contacts)**	**I80 (8 contacts)**	**I80 (11 contacts)**
**Y81 (1 contacts)**	**Y81 (1 contact)**	**Y81 (3 contacts)**
**N82 (5 contacts)**	**N82 (6 contacts)**	**N82 (4 contacts)**
**M85 (20 contacts)**	**M85 (32 contacts)**	**M85 (33 contacts)**
**G86 (4 contacts)**	**G86 (2 contacts)**	**G86 (5 contacts)**
V87 (4 contacts)	——	V87 (3 contacts)
**S88 (8 contacts)**	**S88 (10 contacts)**	**S88 (15 contacts)**
**L90 (10 contacts)**	**L90 (19 contacts)**	**L90 (13 contacts)**
A91 (9 contacts)	——	——
P92 (2 contacts)	——	——
I123 (4 contacts)	——	——
**E125 (10 contacts)**	**E125 (5 contacts)**	**E125 (6 contacts)**
**A127 (8 contacts)**	**A127 (4 contact)**	**A127 (1 contact)**
T128 (8 contacts)	——	T128 (2 contacts)
**F129 (45 contacts)**	**F129 (21 contacts)**	**F129 (32 contacts)**
**P130 (22 contacts)**	**P130 (42 contacts)**	**P130 (18 contacts)**
——	T131 (9 contacts)	T131 (2 contacts)
G132 (2 contacts)	——	——
**N133 (15 contacts)**	**N133 (8 contacts)**	**N133 (5 contacts)**
E135 (8 contacts)	——	——
Q137 (1 contact)	——	——

^a^ The nectin-1 amino acids locating within a distance of 4.5 Å from the bound ligand (PRV gD based on the complex structure reported in the current study, HSV-1 and -2 gDs according to previously reported complex structures of PDB code 3U82 and 4MYW, respectively) were selected, and then listed in a pairwise manner. The number of Van der Waals contacts that each interface residues contribute is summarized in parenthesis. Those amino acids utilized to contact all the three viral ligands were highlighted in boldface.

### Mutagenesis study of the key interface residues in nectin-1

**Fig 6:**
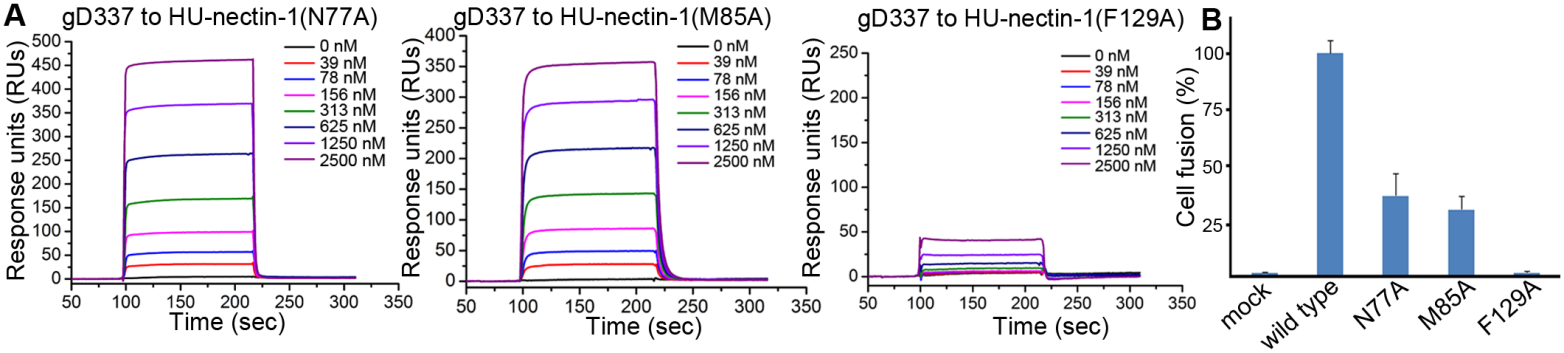
Mutation of the key interface residues in nectin-1 undermines the interaction with PRV gD. (A) SPR tests of the binding between nectin-1 mutants and PRV gD337. The kinetic profiles are recorded and shown. (B) Decreased cell fusion with the mutated nectin-1 receptors. CHO-K1 cells expressing PRV gD/gB/gH/gL and T7 luciferase were mixed and incubated with those expressing T7 polymerase in combination with wild type or mutant HU-nectin-1. The histogram shows the efficiencies of cell fusion with the indicated nectin-1 mutants in comparison to that with the wild type receptor. The results are expressed as means ± SD from three independent experiments.

To further confirm the binding features observed in our complex structure, the key interface residues in nectin-1, including N77, M85 and F129 (which contribute more than 20 hydrophobic vdw contacts each (Table 2) and/or the side-chain H-bonds (Fig 5D), were individually mutated and tested for their interactions with PRV gD337. In the context that HU- and SW-nectin-1 are equally competent in binding with PRV gD (Fig 2B) and their interface residues contacting gD are absolutely conserved (Fig 5E), we therefore utilized the human homolog for the test in our mutagenesis study. In consistent with the structural observation that F129 provides the maximum amount of intermolecule contacts (45 contacts, Table 2), the F129A mutation almost completely abrogated the gD/nectin-1 binding (*K*_d_ > 40 μM) (Fig 6A) and therefore the gD/nectin-1 dependent cell fusion (Fig 6B). The functional indispensability of nectin-1 F129 in PRV-gD engagement was also demonstrated in a previous study[30].

In contrast to F129 with 45 vdw contacts for PRV-gD, the intermolecule binding contributed by nectin-1 N77 (29 contacts) and M85 (20 contacts) were only about half of that for F129 (Table 2). Accordingly, substitution of the two residues with alanine decreased, but not abolished, the gD/nectin-1 interaction. The affinities of the nectin-1 N77A and M85A for gD337 were determined to be 3.4 μM and 2.8 μM, respectively (Fig 6A), and the two mutants remain competent in the gD-mediated cell fusion, though with decreased efficiencies (Fig 6B). It is notable that nectin-1 N77 and M85 interact more extensively with HSV-gD (e. g. 42 and 33 contacts, respectively with HSV-2 gD) than with PRV-gD (29 and 20 contacts, respectively) (Table 2), echoing a previous study reporting their important roles in binding the HSV ligand but much compromised contributions to the engagement of the PRV protein [31]. These mutagenesis data coincide well with our structural observation, which conversely verified the PRV-gD/nectin-1 binding mode observed in the complex structure. It is also noteworthy that nectin-1 N77A and M85A engage PRV-gD with a fast-on/fast-off mode (Fig 6A), forming contrast to the wild type protein which shows both slow association and dissociation rates. Similar phenomena of altered *K*_on_/*K*_off_ rates have also been observed in other virus-ligand/receptor binding pairs when their interface residues are mutated [22].

## Discussion

Of the thus-far identified alphaherpesviral gD receptors, nectin-1 is likely the most effective in terms of its wide usage by different viruses. HSV-1, HSV-2, PRV and BHV-1 are all reported to utilize nectin-1 for cell entry [8, 12, 13, 30, 31]. The molecular basis of nectin-1 recognition by the envelope gD proteins of these viruses, therefore, is an interesting but yet an unresolved issue. Previous studies focused on HSV, a member of the *Simplexvirus* genus in the *Alphaherpesvirinae* subfamily. The structures of both the free HSV gD and its complex with nectin-1 are reported at high resolutions [6, 21, 22, 25]. Nevertheless, the structural and functional features of other alphaherpesviral gDs remains poorly understood. In this study, we have reported the first structure of gD derived from a *Varicellovirus* member of the alphaherpesviruses, the PRV. Despite of its low sequence identity to the HSV homologs (~ 22%), PRV gD reserves the canonical gD features, including an IgV-like core with a kinked C” strand and the surface-exposed N- and C-terminal extensions. We further solved the complex structure of PRV gD bound with SW-nectin-1, which revealed a similar nectin-1 binding mode as observed for HSV gD. Nevertheless, several unique features at the PRV-gD/nectin-1 binding interface (e. g. a bulged α2' helix of PRV gD interacts with an adjusted CC’ loop in nectin-1, an about 3.5-Å shift for PRV gD (relative to HSV gD) when bound to the receptor) suffice the PRV ligand to recognize nectin-1 using quite different gD residues from those of the HSV homologs. These structural observations therefore provide a systematic view on the receptor binding mechanism of a second alphaherpesvirus and yet the first in the *Varicellovirus* genus.

It is interesting that both in the free PRV gD structure and in the PRV-gD/nectin-1 complex structure, a large C-terminal region of the gD molecule are untraceable. We noted that it has been a long time (over four months) before the proteins (gD337 for the free structure and gD284 for the complex structure) were successfully crystallized. Taking into account that large disordered parts of a protein are likely perturbing crystal formation, it may indicate a possible proteolysis of both the gD337 and the gD284 C-terminal regions during crystallization, leading to similar proportion of the gD residues (P7-P250 in the free gD structure and P9-D241 in the complex structure) being successfully traced in the free gD and the nectin-1 bound structures. We therefore believe that the re-orientation of the gD N-loop before and after nectin-1 binding is more likely the result of receptor engagement than arising from the differences in the gD forms used for crystallization. A flexible N-loop which experiences rearrangement or is structurally stabilized upon receptor binding has also been observed for HSV gDs [22, 26].

With an overall similar structure, PRV and HSV gDs all engage the nectin-1 CC'C''FG sheet for receptor recognition. Along the sheet, a total of 20 residues (out of 31 for PRV gD, 22 for HSV-1 gD and 26 for HSV-2 gD) were recognized by all the three viral ligands (summarized in Table 2). It is notable that these amino acids contribute the majority of the inter-molecule interactions and therefore constitute a conserved central contact interface dominating gD-binding, whereas the remaining nectin-1 residues provided additional and supplementary contacts for engagement of gD. It is interesting that the PRV and HSV gDs, with only 22% sequence identity, select essentially the same contact interface in nectin-1 for recognition and engagement. We believe this interface patch likely has evolved as a major ligand-binding entity, dominating the nectin-1 interaction not only with PRV and HSV, but also with other alphaherpesviruses (e. g. BHV-1). It is also noteworthy that it is not rare phenomena that different viruses could recognize the same host surface molecule as the cellular receptor. It would therefore be interesting to investigate if other viruses similarly recognize extensively overlapped surface patches during receptor engagement, as observed for the PRV/HSV pair. To our knowledge, the Middle East respiratory syndrome coronavirus and the related bat-derived HKU4 coronavirus represent another example of such case. Both viruses recognize human CD26 via the viral spike protein, and they were shown to bind to the same propeller elements of the receptor [32, 33].

The PRV gD footprint in nectin-1 also coincides with the receptor dimerization interface. According to the previous studies, formation of the homo- and/or hetero-dimers is the basis for nectin-1 to exert its cell adhesion functions [34]. PRV should therefore compete against the receptor dimerization by gD engagement, via which jeopardizing the host junction architecture. It should be noted that the affinity of PRV gD for HU-nectin-1 is determined to be 191 nM, which is about 100 fold higher than that calculated for nectin-1 self-interactions [23]. This should confer the binding with PRV gD an astonishing priority during the viral infection over nectin-1 dimerization, therefore exploiting its cell adhesion functions.

In HSV, the membrane-proximal loop of the gD ectodomain is proposed as the PFD which would interact with gB/gH/gL to trigger membrane fusion [19]. According to a previously reported dimeric structure of HSV gD, the viral ligand prelocates its PFD in a position that will preclude the binding of gD with its receptors [25]. The gD/receptor engagement therefore would only occur when this prelocated loop is displaced, exposing the otherwise locked receptor binding site. Consistent with this structural observation, a short HSV gD protein lacking PFD is about 100-fold more competent than the long gD form in terms of receptor binding [20, 25]. In the current study, we demonstrated that the affinity of PRV gD284 (a short gD variant without the membrane-proximal loop) for nectin-1 is also dramatically (~12–16 folds) higher than that of gD337 (a long gD form containing the whole ectodomain). We believe this phenomenon echoes what has been observed with HSV-gD PFD, indicating a role of the PRV-gD membrane-proximal loop in membrane fusion. It is also noteworthy that the enhanced receptor binding affinity for the short variant of HSV gD stems mainly from a significantly increased binding on-rate (~ 40 fold in *K*_on_), falling in a good accordance with the prelocation of PFD in gD [25]. In contrast, the changes in *K*_on_ between PRV gD284 and gD337 is only about 5–8 folds. This argues against a prelocation of the membrane-proximal loop in PRV gD and may indicate a novel mechanism of the loop interfering with nectin-1 binding. These are interesting issues that are worth of studying in the future.

In contrast to HSV, both previous studies and the functional data in this report demonstrated that PRV can not use HVEM as a cellular receptor [12, 35]. It is notable that the HVEM binding site in HSV gD is allocated to an N-terminal hairpin which was re-constituted by the long protein N-loop upon HVEM engagement. An N-loop of sufficient size is therefore a prerequisite for the gD ligand to interact with HVEM [27]. Facilitated by the structure-based sequence alignment between PRV and HSV gDs, it is clear that the N-loop of the PRV protein is only about half size of that in HSV gD. We believe this dramatically shortened N-loop in the PRV ligand can not support HVEM-binding, representing the structural basis of its inertness in HVEM recognition.

The PRV infection can cause pseudorabies (also known as Aujeszky's disease) in pigs and other animals [6]. Although the disease can be prophylactically controlled with the live attenuated virus vaccine, it remains a serious problem and potential threat to the swine industry of many countries, leading to heavy economic losses each year. With an indispensable role in the viral infection, PRV gD represents a good vaccine candidate [36–38]. The strategy reported in the current study of preparing recombinant PRV-gD proteins (gD337 and gD284) with both structural integrity (correctly folded as demonstrated with the free and the nectin-1-bound structures) and functional competency (able to engage the receptor as shown with the *in vitro* SPR data) might facilitate the development of a gD-based subunit vaccine in the future.

## Materials and methods

### PRV infection experiment

CHO-K1 cells (a Chinese hamster ovary cell line which is an already-existing collection in the laboratory) were maintained in Dulbecco's minimal essential medium (DMEM) supplemented with 10% fatal bovine serum (FBS), 100 U/ml penicillin, and 100 mg/ml streptomycin. For the PRV infection experiment, cells were seeded in the 6 well plate with 70% confluence. After 24h, cells were transfected with pcDNA4.0-HU-nectin-1, pcDNA4.0-HU-HVEM or pcDNA4.0-SW-nectin-1 expression plasmids, separately. After 24 h, cells were incubated with PRV Bartha-K61 solution (100 TCID_50_/ml PRV in DMEM) at 37°C for 3 h, after which 2% FBS was added to the medium. Cells were then photographed under microscope 72 h post infection.

### Construction of the expression plasmids and preparation of the proteins

DNAs encoding the ectodomains of the human and swine nectin-1 (amino acids 30–335) were obtained by PCR using the primer pairs of human-Nectin1-F (5'- CGCGGATCCGTCCCAGGTGGTCCAGGTGAAC -3') / human-Nectin1-R (5'-AAGGAAAAAAGCGGCCGCTTCTGTGATATTGACCTC-3') and porcine-Nectin1-F (5'-CGCGGATCCGACCCAGGTGGTCCAGGTGAACG-3') / porcine-Nectin1-R (5'-AAGGAAAAAAGCGGCCGCCTCTGTGATATTGACCTCCACC-3'), respectively. The amplified fragments were then individually cloned into the prokaryotic expression vector pET21b (Invitrogen) with the BamHI and NotI sites. The proteins were then prepared following the reported methods [21]. In brief, the proteins were expressed as inclusion bodies in *E*. *coli* BL21 (DE3) and the inclusion bodies were prepared in a buffer composed of 100 mM Tris-HCl, pH 9.0, 400 mM L-Arginine, 2 mM EDTA, 5 mM reduced glutathione and 1 mM oxidized glutathione. Then, the refolded proteins were subjected to gel-filtration on a Hiload 16/60 Superdex 200 column (GE Healthcare). By estimation of their MWs, the monomer peaks could be obtained. The resultant monomeric proteins were carefully collected, concentrated, and then applied for kinetic studies using surface plasmon resonance.

The DNAs coding for PRV (Becker strain) gD ectodomain of residues 1–284 (gD284) and 1–337 (gD337) were amplified by PCR with the same forward primer of PRV-gD-F (5'-CCGGAATTCAGGACGTGGACGCCGTGCC-3') pairing with different reverse primers of PRV-gD284-R (5'-CCGCTCGAGTTAGTGGTGATGGTGGTGGTGCCGCGTCGCCGGCTCGGGCAG-3') and PRV-gD337-R (5'-CCGCTCGAGTTAGTGGTGATGGTGGTGGTGGCGGTGGCGCGAGACGCC-3'), respectively. The coding sequence of the swine nectin-1 IgV domain (amino acids 37–143) was amplified using primers pNectin-1-IgV-F (5'-CCGGAATTCAGGACTCCATGTATGGTTTCATCGGC-3') and pNectin-1-IgV-R (5'-CCGCTCGAGTTAGTGGTGATGGTGGTGGTGCATCACAGTGAGGTTGAGCT-3'). The resultant DNA fragments that contain a C-terminal 6×his coding sequence were then individually cloned, via EcoRI and XhoI sites, into a previously modified pFastBac1 vector, which has been engineered to incorporate a baculovirus gp67 signal sequence at the N-terminus [39–41]. The pFastBac1 construct for HSV-1 (KOS strain) gD (residues 1–285, gD285), which is an already-existing collection in the lab, has been described previously (21). All the proteins were expressed with the Bac-to-Bac baculovirus expression system (Invitrogen). The recombinant baculovirus was used to infect Hi5 cells (a Trichoplusia ni insect cell line which is an already-existing collection in the laboratory) for the production of the soluble proteins. The proteins were purified from the Hi5 cell supernatants first by nickel affinity chromatography (GE Healthcare) and then by gel-filtration chromatography using a Superdex 200 column (GE Healthcare).

### Surface plasmon resonance (SPR) measurements

The mutant proteins of human nectin-1 ectodomain were prepared as previously described [21]. The binding kinetics between the soluble gD and nectin-1 was analysed at 25°C on a BIAcore 3000 machine with CM5 chips (GE Healthcare). HBS-EP buffer (10 mM HEPES, pH7.4, 150 mM NaCl, 3 mM EDTA, 0.005% Tween 20) was used for all measurements. For SPR measurements, both gD and nectin-1 proteins were purified by gel filtration using Superdex 200 column (GE Healthcare). We used the blank channel as negative control. About 1,200 response units of nectin-1 were immobilized on the chip. When the data collection was finished in each cycle, the sensor surface was regenerated with 10 mM NaOH. A serial of concentrations up to 2,500 nM were designed for the experiment.

### Kinetic study

The kinetic analysis was performed by SPR using the BIAcore 3000 system. Gradient concentrations of PRV gD284 and PRV gD337 were flowed at 30 μL/min over nectin-1 (WT or mutant) immobilized at about 1,200 response units, and tested for binding at 25°C. The running buffer is composed of 10 mM HEPES, pH7.4, 150 mM NaCl, 3 mM EDTA, 0.005% Tween 20.

### Crystallization

All the crystals were obtained, with the hang-drop vapor-diffusion method, by initial screening with the commercial Hampton Research kits and then by condition optimizations. Free PRV gD crystal was finally obtained by mixing 1 μl of the concentrated gD337 protein at 10 mg/ml with 1 μL reservoir solution consisting of 0.1 M ammonium acetate, 0.1 M bis-tris pH 5.5, 17% w/v polyethylene glycol 10,000. To obtain the crystal of the PRV gD/SW-nectin-1 IgV complex, two proteins (gD284 and SW-nectin-1 IgV) were separately purified by gel-filtration chromatography, mixed at 1:1 molar ratio, and incubated on ice for 2 h. The mixture was further purified by gel-filtration chromatography using a Superdex 200 column (GE Healthcare) and the complex peak was carefully collected and then the PRV gD/SW-nectin-1 IgV complex was concentrated to 5 mg/mL. Diffractable crystals were finally obtained by mixing 1 μL of the protein complex with 1 μL reservoir solution consisting of 0.2 M sodium chloride, 0.1 M sodium acetate pH 5.5, 20% w/v PEG 10,000, followed by incubation at 4°C for about 4 months.

### Data collection and structure determination

The diffraction datasets were collected at beamline BL19U1 of the Shanghai Synchrotron Radiation Facility (SSRF) using the synchrotron radiation at 100K. The cryoprotectant solution is composed of 15% V/V glycerol and 85% V/V reservoir solution. Data were then processed with HKL2000 [42] for indexing, integration and scaling. Via molecular replacement using the PHASER [43] program in the CCP4 suite [44], the free PRV gD337 and the PRV gD284/ SW-nectin-1 IgV complex structure were solved with HSV2 gD (PDB code 4MYV) and HSV-1 gD285/ human nectin-1 complex structure(PDB code 3U82) as the search models. The coordinates and the related structural factors have been deposited into the Protein Data Bank with the PDB codes of 5X5V for the free PRV gD structure and 5X5W for the PRV-gD/nectin-1 complex structure.

### Cell fusion assay

The fusion mediated by HSV gB/gD/gH/gL and the receptors has been validated in various cell types [24, 29]. In this study, we set up a cell-based fusion system using CHO-K1 cells as previously reported [29]. In brief, the genes of PRV gB, gD, gH, and gL and nectin-1 were cloned into the pcDNA4.0-myc-his vector to yield the respective plasmids for protein expression in mammalian cells. The T7 polymerase and T7 luciferase expression plasmids were constructed previously in our lab. The expressing plasmids for PRV gB, gD, gH, gL and the T7 luciferase or the plasmids for nectin-1 and the T7 polymerase were separately co-transfected into CHO-K1 cells using Lipo2000 (Invitrogen) according to the manufacturer's instructions. After 24 h of transfection, the cells expressing gB/gD/gH/gL and nectin-1 were mixed and incubated for cell fusion. The luciferase activity was tested using a luciferase assay system kit (Promega).
